# Data-Driven Parameter Design of Broadband Piezoelectric Energy Harvester Arrays Using Tandem Neural Networks

**DOI:** 10.3390/mi17020210

**Published:** 2026-02-04

**Authors:** Zhiyan Cai, Rensong Yin, Chong Liu, Lingyun Yao, Rongxing Wu, Hui Chen

**Affiliations:** 1 Center for Mechanics Plus Under Extreme Environments, Ningbo University, Ningbo 315211, China; 2 College of Engineering and Technology, Southwest University, Chongqing 400715, China; 3 Department of Architectural Engineering, Ningbo Polytechnic University, Ningbo 315800, China; 4 Piezoelectric Device Laboratory, Faculty of Mechanical Engineering and Mechanics, Ningbo University, Ningbo 315211, China

**Keywords:** piezoelectric energy harvester, broadband energy harvester arrays, parameter design, tandem neural networks, finite element simulation

## Abstract

Broadband piezoelectric energy harvesters (PEHs) are attractive for powering self-sustained sensing nodes in industrial monitoring, structural health monitoring, and distributed IoT systems, where ambient vibration spectra are often uncertain, drifting, and broadband. However, tuning multiple resonant peaks in PEH arrays usually relies on time-consuming finite element (FE) parameter sweeps or iterative optimizations, which becomes a practical bottleneck when rapid, site-specific customization is required. This study presents a data-driven inverse-design framework for a five-beam PEH array based on a tandem neural network (TNN). A forward multilayer perceptron (MLP) surrogate is first trained using 10,000 COMSOL-generated samples to predict the array’s characteristic frequencies from the design variables (end masses $M_{1}$–$M_{5}$ and tilt angle α), achieving >98% prediction accuracy with a prediction time <1 s, thereby enabling efficient replacement of repeated FE evaluations during design. The trained MLP is then coupled with an inverse-design network to form the TNN, which maps target characteristic-frequency sets directly to physically feasible parameters through the learned surrogate. Multiple representative target frequency sets are demonstrated, and the TNN-generated designs are independently verified by COMSOL frequency–response simulations. The resulting arrays achieve broadband operation, with bandwidths exceeding 10 Hz. By shifting most computational cost to offline dataset generation and training, the proposed spectrum-to-parameter pathway enables near-instant parameter design and reduces reliance on exhaustive FE tuning, supporting rapid, application-specific deployment of broadband PEH arrays.

## 1. Introduction

Energy harvesting converts ambient energy into electrical power and offers a practical pathway toward self-powered low-power devices such as wireless sensor nodes [1,2], wearable electronics [3], and embedded monitoring systems [4,5], where battery replacement can be costly or impractical. In particular, the deployment of Internet of Things (IoT) sensor networks for long-term environmental and infrastructure monitoring imposes stringent requirements on energy autonomy, reliability, and adaptability to varying ambient vibrations [6]. Among common ambient sources, mechanical vibration is widely available in transportation, industrial equipment, and civil infrastructure, making vibration-driven energy harvesting an active research area. Piezoelectric energy harvesters are particularly attractive because they can generate relatively high voltage, they provide high energy density, and they can be miniaturized and integrated with micro-fabrication processes [7,8].

A persistent limitation of conventional linear PEHs is their inherently narrow operating bandwidth: peak output typically occurs near a single resonance, and performance drops sharply when the excitation frequency deviates from that point. In realistic environments, vibrations often have time-varying or broadband spectra, which motivates design methodologies that can broaden the effective operating frequency range without excessive structural complexity or impractical tuning requirements.

To address the bandwidth limitation, existing approaches can be broadly grouped into (i) nonlinear mechanisms and (ii) resonance tuning/tracking strategies [9]. Nonlinear harvesters—often based on bistability, multistability, magnetic interactions, or piecewise stiffness—can exhibit amplitude-dependent frequency responses and potentially broaden the response band under certain operating conditions [10,11]. However, nonlinear designs may introduce additional complexity, parameter sensitivity, and fabrication/integration challenges, and their broadband advantage can depend strongly on excitation levels and system damping. Resonance tuning methods, including adjustable proof masses and adaptive stiffness mechanisms, aim to shift the resonance to match changing excitation frequencies [12,13] but may require additional components, control strategies, or increased system complexity.

A complementary and scalable pathway is array-based broadband harvesting, where multiple cantilever beams are designed with staggered characteristic frequencies so that the aggregate response covers a wider band. In array PEHs, bandwidth broadening can be achieved by distributing end masses across beams and by modifying geometric or configuration parameters (e.g., mass block placement/tilt) to tailor individual resonances and superimpose multiple peaks [14,15]. While effective, array design introduces a practical bottleneck: as the number of design variables increases (e.g., multiple end masses plus configuration angles), the process of forward simulation and inverse tuning becomes high-dimensional, strongly coupled, and time-consuming if performed through repeated finite element method (FEM) sweeps.

This motivates data-driven design approaches that can replace or reduce costly FEM iterations. Recent years have seen rapid progress in surrogate modeling and parameter design using machine learning, particularly for complex multiphysics systems where direct optimization is expensive, such as in solving nonlinear partial differential equations [16,17] and designing elastic wave metamaterials  [18,19,20,21,22,23,24]. In energy-harvesting research, modern studies increasingly combine simulation data with learning-based predictors to accelerate performance estimation and enable efficient optimization under realistic constraints [25,26].

In inverse design of mechanical metamaterials, a variety of data-driven approaches have been explored, including conditional GANs, invertible neural networks (INNs), and surrogate model with iterative optimization frameworks (e.g., Bayesian optimization) [27,28,29]. We adopt a tandem neural network (TNN) mainly because the performance to structure mapping in this problem is inherently a non-unique mapping; directly training an inverse network to regress a single ground-truth structural label can easily lead to overfitting and reduced design diversity. Instead, the TNN is trained in a forward-consistent manner—an inverse network proposes a design, a pre-trained forward model predicts its performance, and the loss is defined as the mismatch between the predicted and target performance—so that the solution is driven by the satisfaction of physical and target criteria rather than the reproduction of a particular training-sample geometry [27]. Moreover, compared with GAN-based methods that may suffer from training instability and limited controllability, as well as Bayesian-optimization-type methods that typically require multiple iterative evaluations for each new target, a TNN concentrates computation in a one-time offline training stage and can generate candidate designs for arbitrary targets via a single, fast inference step. This efficiency advantage—i.e., replacing expensive simulations with a learned surrogate to substantially accelerate optimization/inverse design—has also been quantitatively demonstrated in related studies [30].

In this work, we propose a data-driven inverse-design framework for a broadband piezoelectric energy harvester array using a TNN, building upon the array configuration studied by Kouritem et al. [14]. We choose the TNN formulation because the inverse mapping from target frequency sets to design parameters is inherently non-unique, and the tandem coupling trains the inverse network through a forward-consistency objective using a frozen, differentiable forward surrogate, which stabilizes learning under constraints and avoids iterative FEM-based tuning during inference. The key idea is to first learn an accurate forward surrogate that maps the design variables (end-mass distribution across beams and a configuration/tilt parameter) to the array’s characteristic frequencies using a large FEM-generated dataset, then couple this frozen forward surrogate with an inverse network so that the inverse-prediction mechanism is trained to satisfy target frequency sets while remaining within bounded, physically meaningful design ranges. This tandem coupling provides an efficient spectrum-to-parameter pathway for fast array customization and reduces reliance on repeated FEM trial-and-error tuning.

The remainder of this paper is organized as follows. Section 2 presents the PEH array configuration and the FEM-based dataset generation. Section 3 details the forward surrogate model and the tandem inverse-design architecture, including training strategy and constraint handling, and reports the validation results with discussions on design performance, robustness considerations, and comparison with baseline design approaches. Finally, Section 4 concludes the paper and outlines future extensions such as multi-objective power-based optimization, tolerance robustness analysis, and experimental verification.

## 2. Modeling and Simulation

### 2.1. Theoretical Modeling

Piezoelectric energy harvesters (PEHs) are primarily characterized by their resonance (modal) frequencies, which largely determine their operating performance. When the excitation frequency deviates from the resonant frequency, both the vibration amplitude and the electrical output drop significantly. Therefore, tuning the characteristic frequencies by adjusting structural parameters provides the foundation for improving harvesting efficiency and for realizing broadband responses.

**Figure 1:**
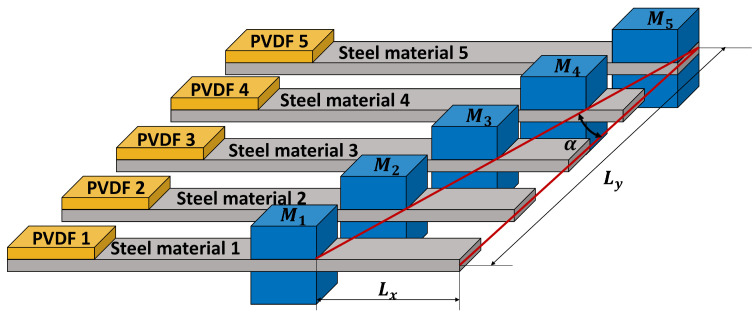
FE model of a five-cantilever PEH array, consisting of steel substrate beams bonded with PVDF patches (PVDF 1–5) and attached end-mass blocks $M_{1}$–$M_{5}$. The mass-block configuration is parameterized by the tilt angle *α*, defined by $\tan\left(\alpha \right)=L_{x}/L_{y}$.

To accurately describe the dynamic behavior of the array adopted in this study, which is a cantilever array with tunable tip masses, a segmented analytical model is established. As shown in Figure 1, each cantilever beam consists of three segments: Segment I ($0\leq x\leq L_{p}$), a composite beam section formed by bonding a piezoelectric layer to an elastic substrate; Segment II ($L_{p}\leq x\leq L_{m}$), a purely elastic substrate beam section; and Segment III ($L_{m}\leq x\leq L_{s}$), and an elastic substrate beam section carrying a tip mass *M*. Here, the position of the proof mass, $L_{m}$, is controlled by the inclination angle *α* (with $\text{tan}\left(\alpha \right)=L_{x}/L_{y}$), which is one of the key design variables for achieving a broadband response from the array.

Based on the classical Euler–Bernoulli beam theory (neglecting shear deformation and rotary inertia), the equations governing the transverse vibration of each segment are as follows [31,32]:

(1) $$-D_{i}\;w_{i,xxxx}\left(x,t\right)=\mu_{i}\;{\ddot{w}}_{i}\left(x,t\right),\;i=1,2,3.$$

where $w_{i}\left(x,t\right)$ is the transverse deflection, $D_{i}$ is the equivalent bending stiffness, and $\mu_{i}$ is the mass per unit length. The subscripts i=1,2,3 correspond to the composite segment, the elastic segment, and the tip-mass tuning segment, respectively (see Appendix A for the detailed segmentation and parameters).

By applying separation of variables, the general solutions of the mode shapes and the dispersion relations can be obtained (see Appendix A for details). Under the boundary conditions at the clamped end (x=0) and the free end ($x=L_{s}$), together with the continuity conditions at the interfaces ($x=L_{p}$ and $x=L_{m}$), the condition for nontrivial solutions can be reduced to the following characteristic equation for the circular frequency *ω*:

(2) $$\det\;\left[H(\omega )\right]=0,$$

where H(ω) is a 12×12 coefficient matrix derived from the boundary and continuity conditions. The smallest positive real root $\omega_{1}$ gives the first natural circular frequency of the system, and the corresponding natural frequency is $f_{1}=\omega_{1}/\left(2\pi \right)$.

This analytical model fully accounts for the piezoelectric composite effect, geometric non-uniformity, and the inertial coupling associated with the tunable tip mass and thus can accurately capture how the stiffness and mass distributions regulate resonance. The high-accuracy analytical results provide a reliable validation benchmark for the finite element model in Section 2.2, thereby ensuring sufficient fidelity of the dataset used for subsequent neural-network training.

### 2.2. Finite Element Model and Validation

A single cantilever-beam piezoelectric energy harvester typically delivers its maximum output only near its characteristic frequency, which restricts performance under variable excitations. To broaden the operational band, multiple cantilever beams can be combined into an array so that their characteristic frequencies are distributed across a target range, thereby producing a broadband response. This array-based broadband strategy has been extensively investigated by Kouritem et al. [14], who used a five-beam array and tuned each beam’s resonance by adjusting the attached end-mass configuration. The present work follows the same array concept and adopts the model framework established in [14]. It is crucial to emphasize that the novelty of this study lies not in this established geometric configuration but in the introduction of a novel data-driven design framework to efficiently determine its optimal parameters ($M_{i}$ and *α*) for target broadband performance. FE simulations are performed in COMSOL Multiphysics 6.2. The FE geometry (Figure 1) consists of five individual cantilever beams with end-mass blocks $M_{1}$–$M_{5}$ attached to the free ends. The position/configuration of the mass blocks is characterized by a mass-block tilt angle *α*, defined by $\tan\left(\alpha \right)=L_{x}/L_{y}$.

The substrate beam is modeled as steel and the piezoelectric layer is modeled as polyvinylidene fluoride (PVDF). The corresponding material properties are provided in Table 1 (e.g., the elastic modulus and density of the steel and the compliance, piezoelectric coefficient, permittivity, and density of the PVDF), and the geometric parameters are summarized in Table 2 (beam length/width and piezoelectric patch dimensions).

To validate the accuracy of the finite element (FE) model, a segmented-beam analytical model established in Section 2.1 was used to investigate the influence of the proof-mass parameters on the first natural frequency of a single cantilever, and the results were compared with the FE predictions.

**Figure 2:**
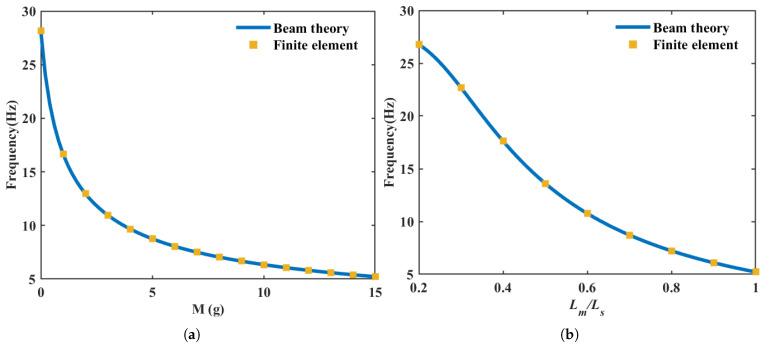
Influence of the proof-mass parameters on the first natural frequency of a single cantilever: comparison between the analytical model and the finite element results. (**a**) The first natural frequency versus proof mass *M* (with $L_{m}$ fixed); (**b**) the first natural frequency versus mass position $L_{m}$ (with M=15g fixed).

Figure 2a shows the variation trend of the first natural frequency with respect to the proof mass *M* (from 0 g to 15 g) when the cantilever thickness is $h_{s}=0.3$ mm and the proof-mass position is set to $L_{m}=L_{p}$. The analytical solutions agree closely with the FE results. Figure 2b further presents, for a fixed mass of M=15 g, the relationship between the frequency and the mass position $L_{m}$ (varying from $L_{p}$ to $0.5L_{p}$), similarly demonstrating a high level of consistency between the two approaches.

These comparisons sufficiently verify the computational accuracy of the FE model in accounting for the effect of the proof mass, thereby providing a reliable numerical basis for the subsequent parametric analysis and data-driven design of the array.

**Figure 3:**
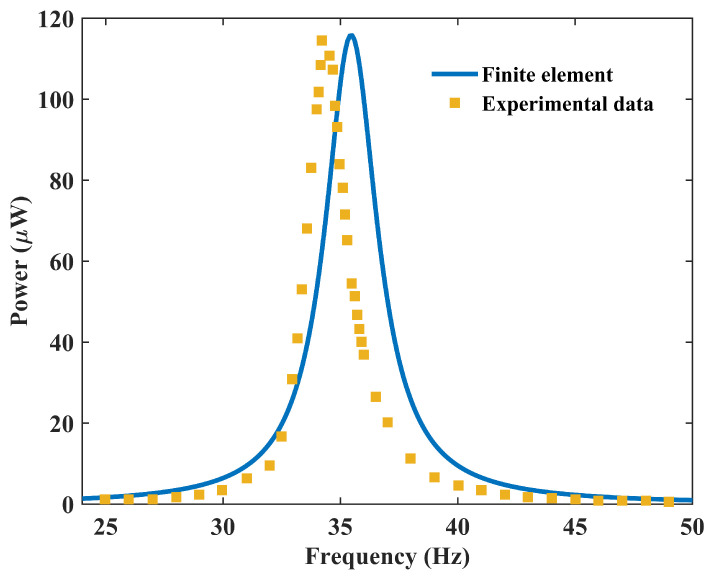
Validation of the finite element model: comparison of the frequency-dependent response of output power under optimum load resistance between a simulation (this work) and experimental data from Song et al. [33].

To further validate the accuracy of the finite element (FE) modeling approach used to generate the complete dataset for this study, we benchmarked it against independent experimental data reported by Song et al. [33] for a PVDF-based energy harvester. Specifically, we replicated the two-layer PVDF cantilever beam structure described in the literature, which features an end mass of 1.872 g. In the simulation, the structure was subjected to the same base excitation acceleration as in the experiment, i.e., 0.5 g, with an isotropic loss factor of 0.07 and an optimal load resistance of R=6.8 MΩ applied across the electrical outputs. As shown in Figure 3, the frequency-dependent response (output voltage/power) predicted by our COMSOL model under these calibrated conditions shows excellent agreement with the measured results. This confirms the reliability of the finite element simulations employed in this study and their ability to accurately capture the electromechanical coupling response of the piezoelectric cantilever beam.

For electrical output evaluation, a resistive load is applied. Per Kouritem et al. [14], the optimal resistance is taken as R=100 MΩ. Under the excitation conditions reported in [14], the frequency response is computed with an excitation amplitude of 3.5 mm, and the output voltage and output power are extracted as the primary performance metrics for subsequent comparisons and for generating the learning dataset used in Section 3. This focus allows us to rigorously validate the TNN framework on a well-defined, high-dimensional parameter optimization problem within a proven structural configuration, while geometric parameters (e.g., beam length and width) are held constant to maintain consistency with the baseline model [14] and to isolate the efficacy of the proposed design method.

### 2.3. Parametric Analysis of Design Variables

In this subsection, we investigate the effects of the end-mass distribution ($M_{1}$–$M_{5}$) and the mass-block tilt angle *α* on the frequency-response characteristics of the five-beam PEH array. All simulations in this study, including those for the parametric analysis in this section and the generation of the 10,000-sample training dataset in Section 3, were conducted under a consistent set of conditions defined by the benchmark study [14]: a base excitation amplitude of 3.5 mm, a load resistance of R=100 MΩ, and a system damping factor of 0.09. Unless otherwise stated, all electrical quantities reported in this section (e.g., voltage and power) refer to peak values.

**Figure 4:**
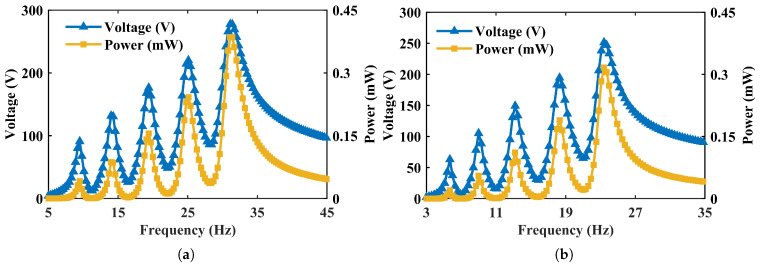
Frequency-dependent output voltage and output power responses of the five-beam PEH array under two end-mass distributions at α=0° (excitation amplitude: 3.5 mm; load resistance: R=100 MΩ). (**a**) Uniform masses $M_{1}=M_{2}=M_{3}=M_{4}=M_{5}=5\;g$. (**b**) Graded masses $M_{1}=15\;g$, $M_{2}=13.5\;g$, $M_{3}=12\;g$, $M_{4}=10.5\;g$, and $M_{5}=10\;g$; mass tuning primarily shifts the characteristic frequencies of individual beams.

To enable a clear comparison of the influence of the tilt angle *α* on the output performance (including voltage, power, and operational bandwidth), we adopted the response of the graded mass distribution shown in Figure 4 (α=0°, $M_{1}$–$M_{5}=15,\;13.5,\;12,\;10.5,\;10\;g$) as the baseline. Its peak voltage and peak power are denoted as $V_{\max}$ and $P_{\max}$, respectively. The voltage and power responses for different values of *α* are then normalized, yielding the normalized voltage $V_{n}=V/V_{\max}$ and normalized power $P_{n}=P/P_{\max}$. This consistent normalization method is also applied to the results of the inversely designed arrays in the following section for a fair performance evaluation.

Figure 4 compares the peak output voltage and peak output power responses under two representative mass configurations at α=0°: (i) a uniform distribution $M_{1}=M_{2}=M_{3}=M_{4}=M_{5}=5\;g$ and (ii) a graded distribution $M_{1}=15\;g$, $M_{2}=13.5\;g$, $M_{3}=12\;g$, $M_{4}=10.5\;g$, and $M_{5}=10\;g$. The results show that changing the end-mass values shifts the operating (characteristic) frequencies of the individual cantilevers significantly, while the influence on the peak values of output voltage and power is comparatively small. This indicates that adjusting $M_{1}$–$M_{5}$ is an efficient and direct lever to allocate the resonance peaks across a desired frequency band in the array configuration.

**Figure 5:**
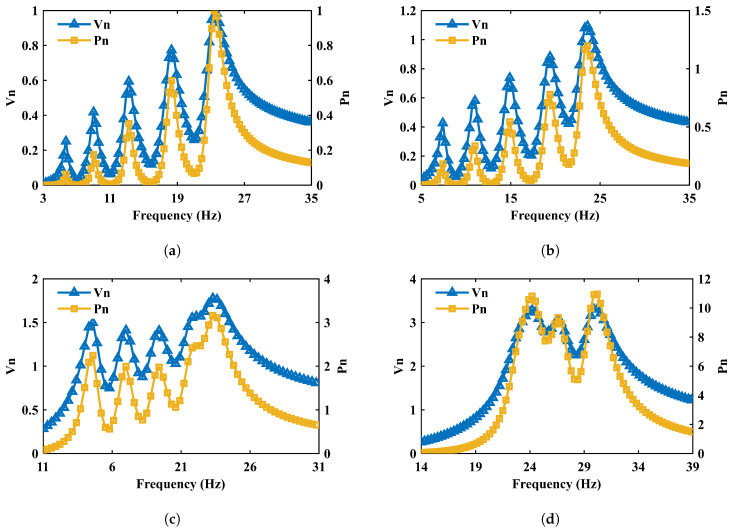
Frequency-dependent output voltage and output power responses of the five-beam PEH array under different mass-block tilt angles α with a fixed end-mass distribution ($M_{1}=15\;g$, $M_{2}=13.5\;g$, $M_{3}=12\;g$, $M_{4}=10.5\;g$, and $M_{5}=10\;g$) (excitation amplitude: 3.5 mm; load resistance: R=100 MΩ). (**a**) α=0°. (**b**) α=14°. (**c**) α=36.8°. (**d**) α=49.3°.

Figure 5 further investigates the influence of the tilt angle *α* while keeping the mass distribution fixed (the graded set above). Four angles are examined: α=0°, 14°, 36.8°, and 49.3°. In contrast to mass tuning, *α* affects not only the locations of the resonance peaks but also the overall levels of output voltage and power. Notably, a pronounced broadband effect emerges when α≥36.8°, and the output power becomes substantially higher than that at α=0°. These trends are consistent with the observations reported in the reference array study, supporting the reliability of the present FE simulations.

The above results demonstrate that the end-mass distribution $M_{1}$–$M_{5}$ primarily controls the allocation of characteristic frequencies, whereas the tilt angle *α* provides a strong additional mechanism for broadband enhancement. Therefore, $M_{1}$–$M_{5}$ and *α* are selected as the key design variables for the data-driven forward prediction and the tandem-network parameter design developed in Section 3.

## 3. Deep Learning Neural Network

Figure 3 and Figure 5 demonstrate that the end-mass distribution $M_{1}$–$M_{5}$ and the mass-block tilt angle *α* have a strong influence on the characteristic frequencies and broadband response of the five-beam array harvester. Accordingly, the design-parameter vector is defined as $x=[M_{1},M_{2},M_{3},M_{4},M_{5},\alpha ]$, and the corresponding characteristic-frequency vector is defined as $f=[F_{1},F_{2},F_{3},F_{4},F_{5}]$. The overall strategy in this section is to first train a fast forward surrogate that approximates the finite element mapping x→f, then construct a constrained inverse-design model that maps target frequencies f→x without requiring expensive FE evaluations during optimization.

### 3.1. Forward-Prediction Network for Predicting Operating Frequency

**Figure 6:**
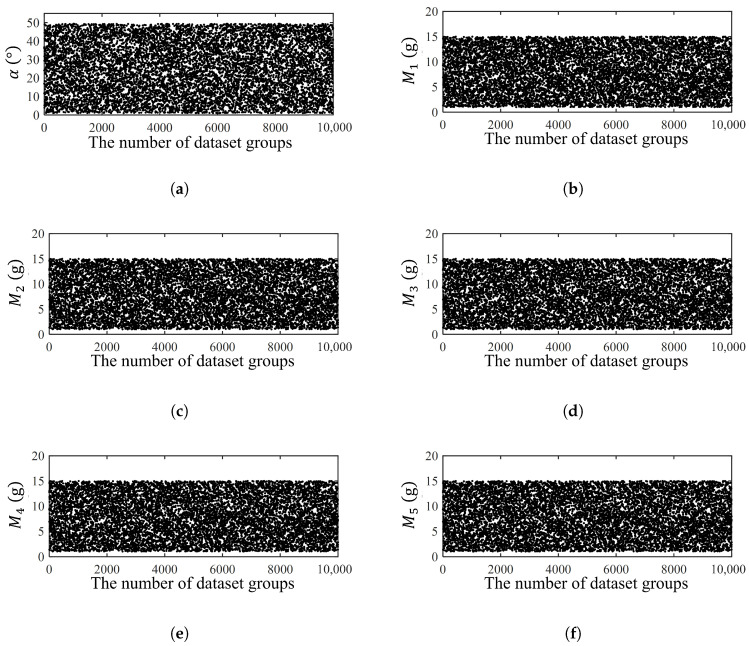
Distributions of the uniformly generated training samples (N=10,000). The design variables were sampled using uniform random sampling within the admissible ranges α∈[0°,49°] and $M_{i}\in \left[1,15\right]\;g$ (i=1–5). Subplots show the sampled values of (**a**) α, (**b**) $M_{1}$, (**c**) $M_{2}$, (**d**) $M_{3}$, (**e**) $M_{4}$, and (**f**) $M_{5}$ versus the dataset index, confirming adequate coverage of the design space.

To enable fast parameter design of the array energy harvester, we first construct a forward surrogate to replace repeated FE simulations. In the inverse-design context, the target is a prescribed characteristic-frequency set, while the design variables are the five end-mass values ($M_{1}$–$M_{5}$) and the mass-block tilt angle (*α*). Embedding FE simulations in the learning loop would require running COMSOL at each iteration to obtain the corresponding operating frequencies, which is computationally inefficient. Therefore, we generate a simulation dataset using COMSOL and train a neural-network-based forward-prediction model to approximate the FE mapping from design variables to characteristic frequencies. Once trained, this model provides a low-cost and differentiable forward simulator for subsequent parameter design. To generate the training dataset, the six design variables $x=[M_{1},M_{2},M_{3},M_{4},M_{5},\alpha ]$ were sampled within the bounded design space α∈[0°,49°] and $M_{i}\in \left[1,15\right]\;g$. Each variable was independently drawn from a uniform distribution over its allowed range, and each sampled design point was evaluated by COMSOL to obtain the corresponding characteristic-frequency vector $f=[F_{1},F_{2},F_{3},F_{4},F_{5}]$. Figure 6 visualizes the resulting 10,000 samples and shows that all variables span the full prescribed ranges without obvious clustering, indicating adequate coverage of the design space for training the surrogate model.

**Figure 7:**
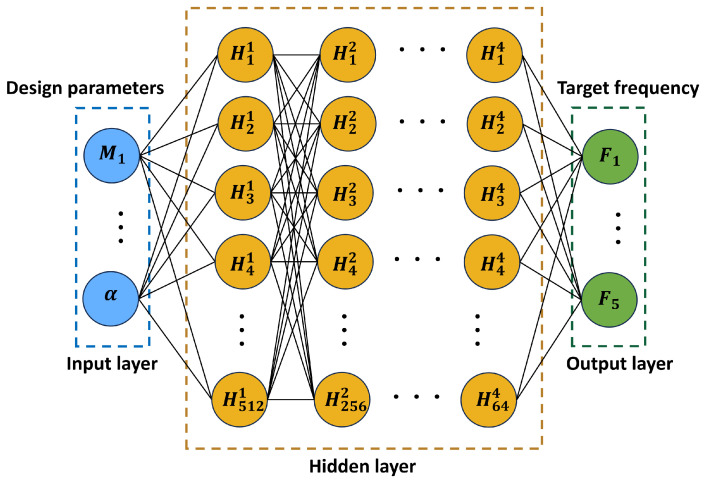
Architecture of the forward-prediction MLP surrogate used to predict the characteristic frequencies of the five-beam PEH array. The inputs are the six design variables (end masses $M_{1}$–$M_{5}$ and mass-block tilt angle *α*), and the outputs are the predicted characteristic frequencies $F_{1}$–$F_{5}$. The MLP consists of four fully connected hidden layers with 512, 256, 128, and 64 neurons, respectively. In the schematic, $H_{j}^{l}$ denotes the *j*th neuron in the *l*th hidden layer, where the superscript *l* indicates the hidden-layer index and the subscript *j* indicates the neuron index (i.e., the total number of neurons in layer *l* is given by the maximum *j* shown). The trained MLP is later used as the forward-simulation module in the tandem neural network (TNN) inverse-design framework.

**Figure 8:**
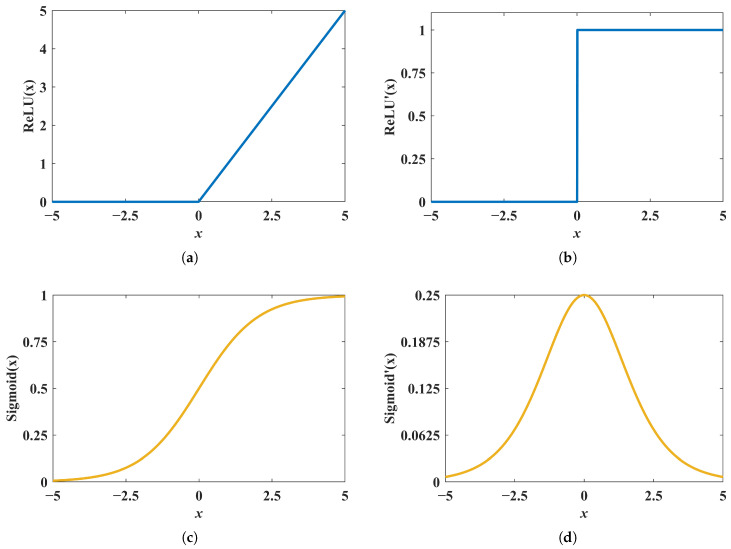
Activation functions used in the forward-prediction MLP. (**a**) ReLU(x), (**b**) derivative of ReLU(x), (**c**) Sigmoid(x), and (**d**) derivative of Sigmoid(x). A ReLU is employed in the hidden layers to improve training stability and mitigate gradient vanishing/explosion, while a sigmoid function is used at the output layer to bound the normalized predictions within [0,1].

An MLP is adopted as the forward-prediction network because it can approximate complex nonlinear relationships using stacked fully connected layers. As shown in Figure 7, the network input is the six-dimensional design vector $\left(M_{1},M_{2},M_{3},M_{4},M_{5},\alpha \right)$, where $M_{1}$–$M_{5}$ denote the end-mass values of the five cantilever beams and *α* characterizes the mass-block configuration. The network output is the five-dimensional frequency vector $(F_{1}$–$F_{5})$, representing the characteristic frequencies of the array harvester. Nonlinear activation functions are required to enhance representational capacity and ensure stable training. A ReLU is used in the hidden layers to mitigate gradient vanishing/explosion and improve convergence in deep fully connected architectures, whereas a sigmoid function is used at the output layer to constrain predictions to [0,1] after normalization. Figure 8 illustrates the function curves and derivatives of ReLU(x) and Sigmoid(x), clarifying their respective roles in the network.

**Figure 9:**
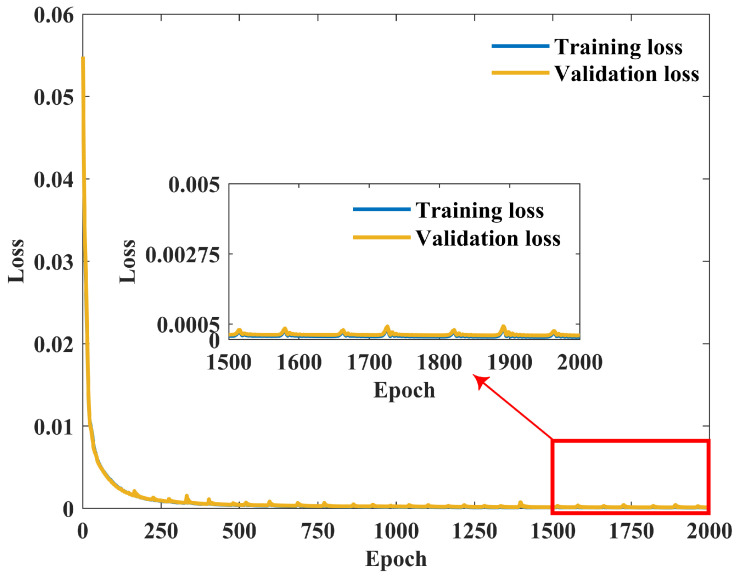
Learning curve of the forward-prediction MLP, showing the training and validation loss (MSE) versus epoch. Both losses decrease rapidly and converge to values below $5\times 10^{-4}$ within the first 250 epochs, after which they remain stable for the rest of the 2000-epoch training, indicating stable convergence of the surrogate model.

The forward network is trained using supervised learning on a dataset generated from FE simulations. The dataset contains 10,000 samples and is split as follows: 80% for training, 10% for validation, and 10% for testing. The learning objective is to minimize the mean squared error (MSE) between predicted frequencies and FE ground truth:

(3) $$L=\frac{1}{n}\sum_{i=1}^{n}{\left({\hat{y}}_{i}-y_{i}\right)}^{2},$$

where *L* is the loss, *n* is the output dimension (n=5), $y_{i}$ is the FE value of the *i*th output frequency, and ${\hat{y}}_{i}$ is the corresponding network prediction. The forward MLP is implemented in TensorFlow and trained for 2000 epochs using the Adam optimizer with a learning rate of $1\times 10^{-3}$. The network architecture comprises five fully connected layers with neuron counts of 512, 256, 128, 64, and 5, respectively. Batch normalization is applied after each hidden layer, and ReLU activation is used in the hidden layers, while the output layer uses a sigmoid activation function to constrain predictions to the normalized range [0,1]. The batch size is set to the full training set per iteration because each design configuration is unique. Early stopping is not applied, as the training converged stably within 2000 epochs, as shown in the learning curve (Figure 9).

**Figure 10:**
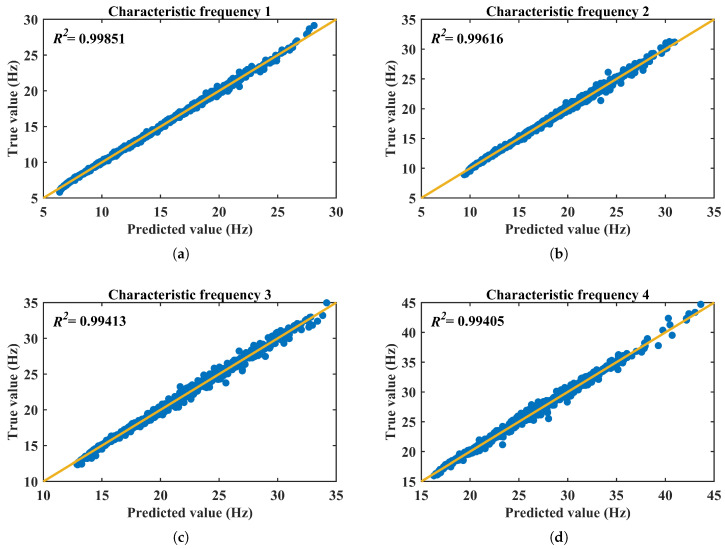

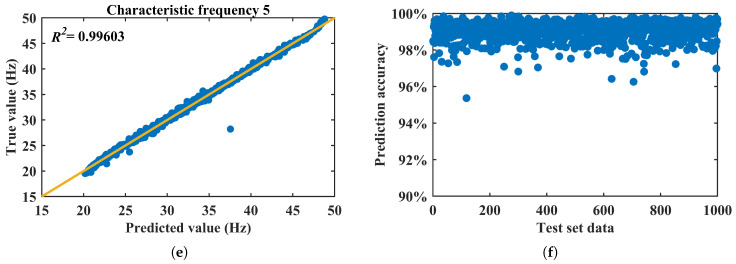
MLP prediction results. (**a**) Characteristic frequency of beam 1. (**b**) Characteristic frequency of beam 2. (**c**) Characteristic frequency of beam 3. (**d**) Characteristic frequency of beam 4. (**e**) Characteristic frequency of beam 5. (**f**) Overall prediction accuracy of the test-set data.

To evaluate prediction performance on unseen samples, the coefficient of determination ($R^{2}$) is used:

(4) $$R^{2}=1-\frac{\sum_{i=1}^{n}{\left(y_{i}-{\hat{y}}_{i}\right)}^{2}}{\sum_{i=1}^{n}{\left(y_{i}-\bar{y}\right)}^{2}},$$

where $\bar{y}$ is the mean of the true values. Figure 10 reports the test-set verification results. Figure 10a–e compare the predicted and true characteristic frequencies for beam 1 to beam 5, respectively, using the 1000 samples in the test set. In each figure, the data points cluster closely around the diagonal line, indicating strong agreement between the MLP predictions and the FE results. The reported $R^{2}$ values are consistently high for all five outputs (approximately 0.994–0.999), confirming that the forward model captures the nonlinear mapping from $(M_{1}$–$M_{5},\alpha )$ to $(F_{1}$–$F_{5})$ with high fidelity. Figure 10f summarizes the overall prediction accuracy across the 1000 test samples; the manuscript reports that the test-set accuracy exceeds 90% and that the inference time is less than 1 s. These results demonstrate that the forward-prediction MLP can reliably replace repeated FE evaluations and provide the foundation for the constrained inverse-design framework introduced in the next subsection.

### 3.2. Constrained Parameter Design Using a TNN and FE Verification

Although the forward-prediction MLP in Section 3.1 can accurately predict the characteristic frequencies of the array harvester, using it for parameter design by directly updating the design variables through gradient descent may lead to unsatisfactory or even incorrect results. The main reason is that, without constraints, the optimized design parameters can drift beyond the learning domain of the MLP, where the surrogate model may misjudge the frequency response. Therefore, constraints must be imposed on the design variables to prevent the optimization from “falling into the wrong area.” In the present work, this is achieved by connecting an inverse-design network to the trained forward-prediction network so that the inverse network’s output range is naturally limited by its output-layer activation, while still enabling end-to-end training through a frequency-mismatch loss. This coupled structure is referred to as a tandem neural network (TNN) [34].

**Figure 11:**
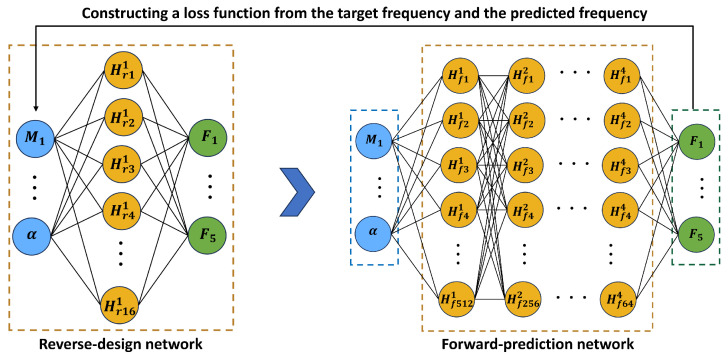
TNN framework for constrained parameter design of the five-beam PEH array. The target characteristic-frequency set $\left(F_{1}–F_{5}\right)$ is input to the reverse (inverse-design) network to generate candidate design parameters $\left(M_{1}–M_{5},\alpha \right)$, which are then fed into the trained forward-prediction MLP to obtain the predicted frequencies. The mismatch between predicted and target frequencies defines the loss used to update *only* the reverse network, while the forward network remains fixed. In the hidden layers, $H_{rj}^{l}$ and $H_{fj}^{l}$ denote the *j*th neuron in the *l*th hidden layer of the reverse and forward networks, respectively (superscript: layer index; subscript letter: network type; subscript number: neuron index). The inverse-network output activation/scaling enforces bounded, physically feasible parameters.

Figure 11 illustrates the complete TNN pipeline for parameter design. The target operating-frequency set (i.e., the set of desired characteristic frequencies) is first fed into the inverse-design network. The inverse network outputs a candidate parameter set (mass-block values and tilt angle). These predicted parameters are then passed into the forward-prediction network trained in Section 3.1, which outputs the corresponding predicted operating frequencies. The predicted frequencies and the target frequencies together form the loss function. During training, only the inverse-design network is updated: it continuously optimizes its internal weights and biases to minimize the frequency mismatch, while the forward-prediction network acts as a fixed surrogate evaluator. In this way, the parameter design remains data-supported and bounded but can still be optimized efficiently using backpropagation.

The inverse network is implemented in TensorFlow and trained using the Adam optimizer with an initial learning rate of $3\times 10^{-3}$ and an exponential decay schedule (decay rate of 0.8 every 5000 steps). The network architecture comprises one hidden layer with 16 neurons, using tanh activation, followed by an output layer with six neurons (corresponding to the five end masses and the tilt angle) with Sigmoid activation to constrain outputs to the normalized range [0,1]. The training is performed for 100,000 epochs with a batch size of one (each target frequency set is processed individually). The forward-prediction MLP remains fixed during this process, and only the inverse network weights are updated to minimize the mean squared error between the target and predicted characteristic frequencies.

**Figure 12:**
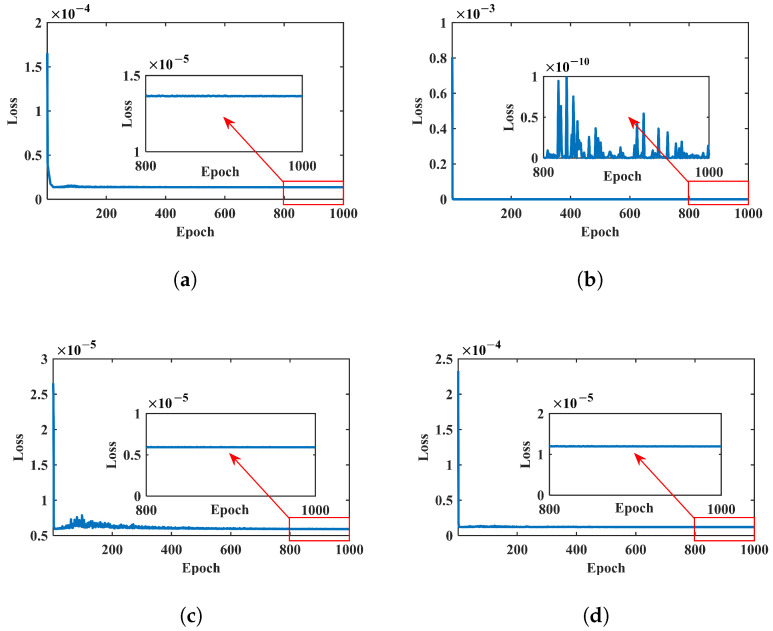
Convergence (loss) curves of the TNN inverse-design training for four target characteristic-frequency sets: (**a**) Group A (26.121, 26.457, 29.404, 30.601, 34.887) Hz; (**b**) Group B (26.688, 28.361, 29.994, 31.639, 33.568) Hz; (**c**) Group C (23.572, 24.776, 25.599, 29.136, 30.332) Hz; and (**d**) Group D (22.349, 24.123, 24.243, 26.292, 28.356) Hz. The rapid decrease in the loss indicates efficient convergence of the constrained inverse-design process.

Figure 12 shows the convergence (loss) curves of the TNN inverse-design training for four representative target characteristic-frequency sets (Groups A–D). The rapid decrease in the loss indicates efficient convergence of the constrained inverse-design process for all target sets. After convergence, the corresponding inverse-designed parameters are obtained. For example, the solution for Group D yields Design B with $\alpha =34.2^{{}^{\circ}}$ and masses $M_{1}$–$M_{5}=2.63\;g$, 4.04 g, 6.12 g, 6.16 g, and 6.17 g.

**Figure 13:**
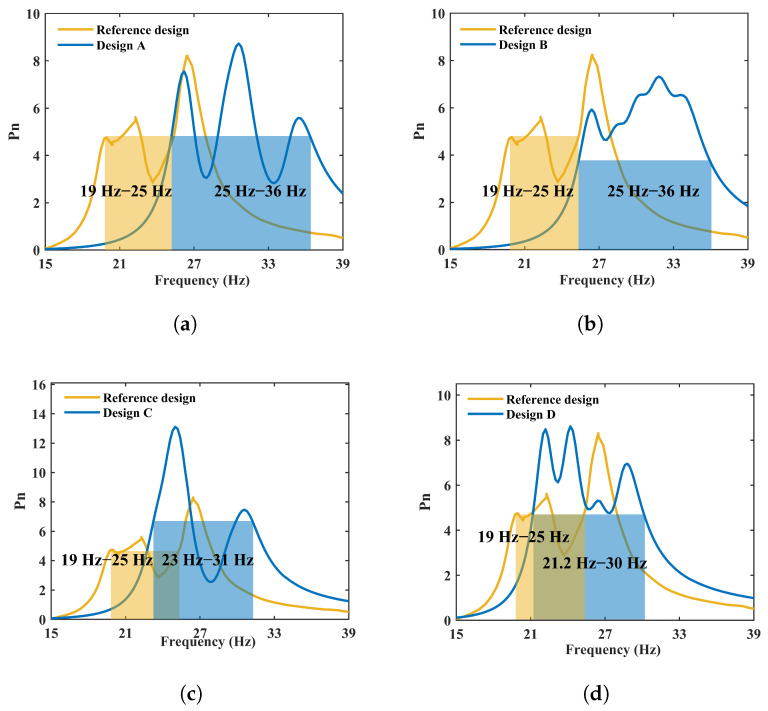
Direct performance comparison between TNN-generated designs and a reference broadband-optimized array. Finite element simulated frequency-dependent output-power responses for: (**a**) Design A from this work (solid blue line; α=49.16°, $M_{1}$–$M_{5}$ = 9.37, 3.16, 9.66, 6.12, 7.49 g) compared to the reference design from Kouritem et al. [14] optimized for maximum contiguous bandwidth (dashed orange line; α=49.3°, $M_{1}$–$M_{5}=15$ g, 13.5 g, 12 g, 10.5 g, 10 g); (**b**) Design B from this work (solid blue line; α=44.96°, $M_{1}$–$M_{5}=6.34,3.29,5.03,6.13,3.96$ g) compared to the same reference design (dashed orange line); (**c**) Design C from this work (solid blue line; α=45.50°, $M_{1}$–$M_{5}=9.69,10.03,4.99,5.76,9.53$ g), compared to the same reference design (dashed orange line); and (**d**) Design D from this work (solid blue line; α=44.71°, $M_{1}$–$M_{5}=14.83,5.52,9.55,7.45,8.93$ g) compared to the same reference design (dashed orange line). The bandwidth is defined as the −3 dB bandwidth (shaded region).

The COMSOL results confirm that the TNN can accurately generate the array parameters needed to realize the desired broadband response; for the four design cases, the resulting −3 dB bandwidth is approximately 10 Hz, demonstrating that the proposed TNN-based inverse-design strategy produces physically valid broadband array PEH designs when validated by FE analysis. Notably, the performance of these TNN-generated designs (see Figure 13) compares favorably with the optimized design from [14], which represents the state of the art achieved through an exhaustive parameter sweep. This demonstrates that our data-driven inverse design framework is not only efficient but also capable of synthesizing high-performance solutions that match or surpass those found by traditional, computationally expensive optimization methods.

To further verify the generalizability of the TNN within the trained design domain, we performed an additional inverse-design test on 20 randomly generated target-frequency sets. The results (detailed in Appendix B) show that the model maintains good inverse-prediction accuracy ($R^{2}>0.91$) over most of the domain, with a slight error increase only for a few designs whose parameters lie near the domain boundaries. This boundary-region performance can be further improved in future work by augmenting training samples near the limits or by adaptive sampling strategies.

## 4. Conclusions

This study demonstrates that the end-mass distribution $M_{1}$–$M_{5}$ and the tilt angle *α* serve distinct and complementary functions in broadband PEH array design: the masses primarily dictate the allocation of resonant frequencies, while the tilt angle is key to enhancing the bandwidth and output level through modified system dynamics, consistent with prior physical insights [14]. Building on this understanding, this paper develops a data-driven inverse-design workflow for a five-beam PEH array using a tandem neural network (TNN). A forward MLP surrogate is trained using 10,000 FE-generated samples to learn the mapping from the design variables $\left(M_{1}\;-\;M_{5},\alpha \right)$ to the characteristic frequencies, achieving high predictive accuracy and sub-second inference. Building on the trained surrogate, an inverse-design network is coupled with the frozen forward network to form the TNN, enabling direct spectrum-to-parameter design under bounded and physically feasible ranges. For two representative target frequency groups, the TNN converges rapidly and produces corresponding mass/tilt designs, and independent FE simulations verify that the designed arrays achieve broadband frequency responses with bandwidths exceeding 10 Hz. From a practical standpoint, this spectrum-to-parameter pathway enables rapid customization of array harvesters for site-specific and time-varying vibration spectra, which is particularly valuable for deploying self-powered sensing nodes where the dominant excitation frequencies are uncertain or drifting.

In contrast to traditional FEM-driven design workflows (e.g., manual parameter sweeps or iterative optimization that repeatedly calls high-fidelity FE simulations for each target), the proposed TNN framework shifts most of the computational cost to an offline dataset-generation/training stage and then avoids repeated FE evaluations during parameter design. As a result, candidate designs can be generated efficiently through the differentiable surrogate and backpropagation, while the final designs remain reliable because they are constrained within the admissible parameter bounds and are independently validated by COMSOL simulations. This combination of computational efficiency and verified design quality highlights the practical value of the proposed approach for rapid customization of PEH arrays under varying target spectra, substantially reducing engineering time and dependence on trial-and-error FEM tuning. In future practical developments, the proposed workflow can be integrated into an engineering design loop in which field-measured vibration spectra are first translated into target characteristic-frequency sets, followed by near-instant parameter generation via the trained TNN and a small number of FE or experimental checks for final verification. Such an approach can significantly shorten the design cycle for broadband PEH arrays and facilitate scalable, application-specific deployment (e.g., for machine-condition monitoring, structural health monitoring, and distributed IoT sensor networks) without relying on exhaustive parameter sweeps.

The current approach is limited by the predefined training domain (α∈[0°,49°] and $M_{i}\in \left[1,15\right]\;g$), and parameter designs outside the learned ranges may degrade in accuracy; moreover, the present parameter design targets characteristic frequencies using mass/tilt parameters only. Future work will incorporate power-oriented objectives (including load/circuit matching), evaluate robustness under manufacturing tolerances (e.g., perturbations in $M_{i}$ and *α*), extend the design space to additional geometric variables (e.g., beam length/width and patch layout), and include experimental validation on fabricated prototypes; multi-objective formulations (e.g., multi-band power targets) will also be explored to enable application-specific broadband and high-efficiency designs. In addition, integrating the inverse-designed PEH arrays with power-management electronics and storage modules and coupling the framework with in situ spectral identification may further enable practical system-level prototypes that deliver more stable power under realistic broadband and nonstationary vibrations.

## Figures and Tables

**Table 1:** Material properties of the substrate (steel) and piezoelectric layer (PVDF) used in the finite element model.

Material Properties (Steel)		Material Properties (PVDF)	
*E*	200 GPa	$s_{11}$	$3.781\times 10^{-10}m^{2}/N$
ν	0.3	$d_{31}$	$1.358\times 10^{-11}\;C/N$
$\rho_{s}$	7850 kg/m^3^	$\varepsilon_{33}$	$7.74\varepsilon_{0}$
		$\rho_{p}$	1780 kg/m^3^

**Table 2:** Geometric parameters of the single cantilever harvesters used in the FE model, including the steel substrate dimensions $\left(L_{s},b,h_{s}\right)$ and the PVDF patch dimensions $\left(L_{p},b,h_{p}\right)$.

Steel		PVDF	
Length ($L_{s}$)	95 mm	Length ($L_{p}$)	15 mm
Width (*b*)	10 mm	Width (*b*)	10 mm
Thickness ($h_{s}$)	0.3:0.1:0.7 mm	Thickness ($h_{p}$)	0.3 mm

**Figure A1:**
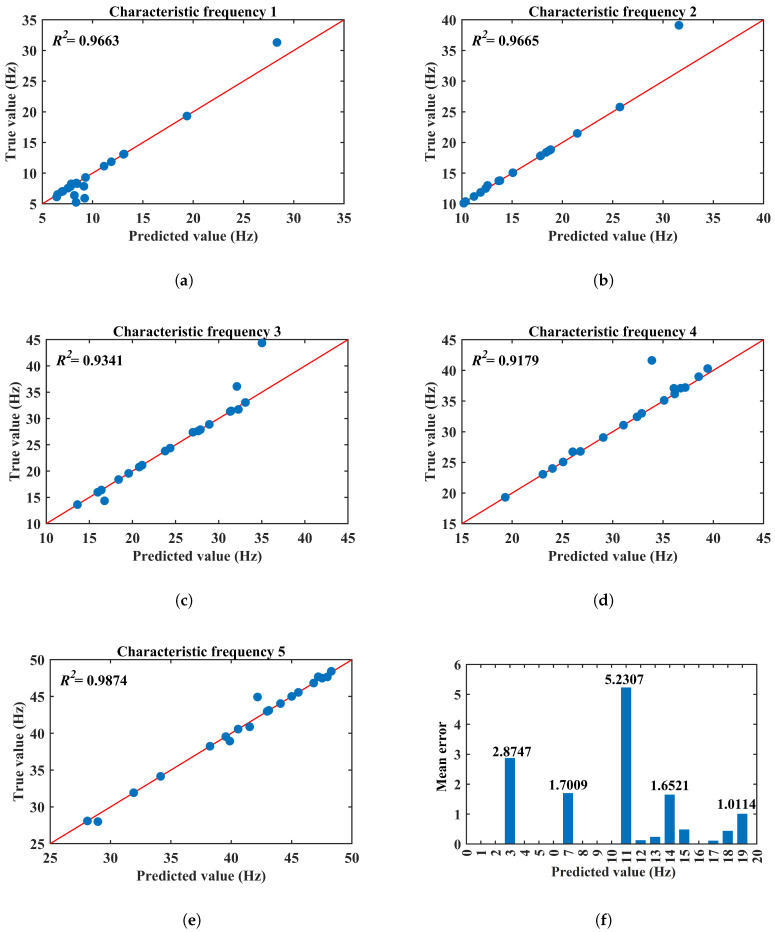
Validation results of the TNN inverse design for 20 random target-frequency sets: (**a**–**e**) predicted versus target characteristic frequencies for beams 1–5, respectively ($R^{2}>0.91$ for all sets); (**f**) mean absolute error (MAE) of each design set.

## Data Availability

The original contributions presented in this study are included in the article. Further inquiries can be directed to the corresponding authors.

## Appendix A. First-Order Natural Frequency of a Single Energy Harvester

For the classical Euler–Bernoulli beam theory, the displacement field is

(A1) $$u=-z\;w_{,x},$$

(A2) w=w(x,t),

where *u* is the axial displacement in the *x*-direction and *w* is the transverse deflection of the beam. Based on the one-dimensional constitutive assumption of the piezoelectric layer, the constitutive relations are

(A3) $$S_{1}^{p}=s_{11}^{E}T_{1}^{p}+d_{31}E_{3},$$

(A4) $$D_{3}=d_{31}T_{1}^{p}+\varepsilon_{33}^{T}E_{3},$$

where $S_{1}^{p}=-z\;w_{,xx}$ is the strain of the piezoelectric layer in the *x*-direction, $T_{1}^{p}$ is the stress (along *x*) in the piezoelectric layer, $E_{3}$ is the electric field in the *z*-direction, and $D_{3}$ is the electric displacement in the *z*-direction. $s_{11}^{E}$, $d_{31}$ and $\varepsilon_{33}^{T}$ denote the elastic compliance, piezoelectric constant and permittivity, respectively. For the elastic layer, the constitutive relation is

(A5) $$T_{1}^{s}=E\;S_{1}^{s},$$

where $S_{1}^{s}=-z\;w_{,xx}$ is the strain of the elastic layer in the *x*-direction and *E* is Young’s modulus.

For the structure shown in Figure 1, when $0<x<L_{p}$, the cantilever is a composite beam made of a piezoelectric layer and an elastic substrate. When $L_{p}<x<L_{s}$, it consists of the elastic substrate and a proof mass; the mass position can be tuned within $L_{p}<x<L_{s}$ (controlled by the angle). Assume the proof mass is located at $x=L_{m}$. Hence, the unit-cell beam is divided into three segments: Segment I ($x\in [0,L_{p}]$), Segment II ($x\in [L_{p},L_{m}]$), and Segment III ($x\in [L_{m},L_{s}]$).

For Segment I, let *a* be the distance from the neutral axis to the bottom surface of the beam, $h_{s}$ the thickness of the elastic layer, $h_{p}$ the thickness of the piezoelectric layer, and *b* the beam width. By the definition of the neutral axis, the resultant axial force on the cross-section is zero:

(A6) $$\int_{-a}^{-(a-h_{s})}T_{1}^{s}\;b\;dz+\int_{-(a-h_{s})}^{h_{p}-\left(a-h_{s}\right)}T_{1}^{p}\;b\;dz=0.$$

Solving (A6) gives

(A7) $$a=\frac{Eh_{s}^{2}+2s_{11}^{-1}h_{s}h_{p}+s_{11}^{-1}h_{p}^{2}}{2Eh_{s}+2s_{11}^{-1}h_{p}}.$$

The bending moment acting on the beam can be written as

(A8) $$M_{1}\left(x,t\right)=\int_{-a}^{h_{p}-\left(a-h_{s}\right)}T_{1}\;b\;z\;dz.$$

Substituting (A3) and (A5) into (A8) yields

(A9) $$M_{1}\left(x,t\right)=-D_{1}w_{,xx}+s_{11}^{-1}d_{31}b\frac{V}{h_{p}}\left(\frac{h_{p}^{2}}{2}-h_{p}a+h_{p}h_{s}\right),$$

where $V=-E_{3}h_{p}$ is the voltage between the two electrodes (an electrode short circuit corresponds to V=0), and $D_{1}$ is the bending stiffness:

(A10) $$D_{1}=bE\frac{{\left(a-h_{s}\right)}^{3}+a^{3}}{3}+bs_{11}^{-1}\frac{{\left(h_{p}-a+h_{s}\right)}^{3}+{\left(a-h_{s}\right)}^{3}}{3}.$$

Substituting (A9) into the beam governing equation gives

(A11) $$-D_{1}w_{1,xxxx}=\mu_{1}{\ddot{w}}_{1}.$$

A general harmonic solution of (A11) is

(A12) $$w_{1}\left(x,t\right)=\left[A_{1}\sin\left(\beta_{1}x\right)+A_{2}\cos\left(\beta_{1}x\right)+A_{3}\sinh\left(\beta_{1}x\right)+A_{4}\cosh\left(\beta_{1}x\right)\right]e^{i\omega t},$$

where $A_{1}$–$A_{4}$ are constants, $\beta_{1}$ is the wavenumber, *ω* is the circular frequency, and *t* is time. Substituting (A12) into (A11) yields

(A13) $$\omega =\beta_{1}^{2}\sqrt{\frac{D_{1}}{\mu_{1}}},$$

where $\mu_{1}=\rho_{s}A_{s}+\rho_{p}A_{p}$ is the mass per unit length of the composite segment, while $A_{s}=h_{s}b$ and $A_{p}=h_{p}b$ are the cross-sectional areas of the elastic and piezoelectric layers, respectively.

For Segment II, the structure is purely elastic, with bending stiffness

(A14) $$D_{2}=\frac{bh_{s}^{3}E}{12},$$

and mass per unit length $\mu_{2}=\rho_{s}A_{s}$. The governing equation is

(A15) $$-D_{2}w_{2,xxxx}=\mu_{2}{\ddot{w}}_{2},$$

whose harmonic solution is

(A16) $$w_{2}\left(x,t\right)=\left[B_{1}\sin\left(\beta_{2}x\right)+B_{2}\cos\left(\beta_{2}x\right)+B_{3}\sinh\left(\beta_{2}x\right)+B_{4}\cosh\left(\beta_{2}x\right)\right]e^{i\omega t},$$

and

(A17) $$\omega =\beta_{2}^{2}\sqrt{\frac{D_{2}}{\mu_{2}}}.$$

For Segment III, the material parameters are the same as those of Segment II; hence, $D_{3}=D_{2}$ and $\mu_{3}=\mu_{2}$. The governing equation is

(A18) $$-D_{3}w_{3,xxxx}=\mu_{3}{\ddot{w}}_{3},$$

with solution

(A19) $$w_{3}\left(x,t\right)=\left[C_{1}\sin\left(\beta_{3}x\right)+C_{2}\cos\left(\beta_{3}x\right)+C_{3}\sinh\left(\beta_{3}x\right)+C_{4}\cosh\left(\beta_{3}x\right)\right]e^{i\omega t},$$

where $\beta_{3}=\beta_{2}$.

For the cantilever beam, the boundary conditions are

(A20) $$w_{1}\left(0\right)=0,\;w_{1,x}\left(0\right)=0,\;D_{3}w_{3,xx}\left(L_{s}\right)=0,\;D_{3}w_{3,xxx}\left(L_{s}\right)=0.$$

The continuity conditions at $x=L_{p}$ are

(A21) $$W_{1}\left(L_{p}\right)=W_{2}\left(L_{p}\right),\;W_{1,X}\left(L_{p}\right)=W_{2,X}\left(L_{p}\right),\;M_{1}\left(L_{p}\right)=D_{2}W_{2,Xx}\left(L_{p}\right),\;M_{1,X}\left(L_{p}\right)=D_{2}W_{2,Xxx}\left(L_{p}\right).$$

The continuity conditions at $x=L_{m}$ are

(A22) $$w_{2}\left(L_{m}\right)=w_{3}\left(L_{m}\right),\;w_{2,x}\left(L_{m}\right)=w_{3,x}\left(L_{m}\right),\;D_{2}w_{2,xx}\left(L_{m}\right)=D_{3}w_{3,xx}\left(L_{m}\right),\;D_{3}w_{3,xxx}\left(L_{m}\right)-D_{2}w_{2,xxx}\left(L_{m}\right)=-M{\ddot{w}}_{2}\left(L_{m}\right).$$

Substituting (A12), (A16) and (A19) into (A20)–(A22) yields a homogeneous linear system for $A_{1},A_{2},\ldots ,C_{4}$:

(A23) $$\left[\begin{array}{ccc} H_{11} & 0 & H_{13}\\ H_{21} & H_{22} & 0\\ 0 & H_{32} & H_{33} \end{array}\right]\left[\begin{array}{c} X_{1}\\ X_{2}\\ X_{3} \end{array}\right]=0$$

where $X_{1}={\left[A_{1},A_{2},A_{3},A_{4}\right]}^{T}$, $X_{2}={\left[B_{1},B_{2},B_{3},B_{4}\right]}^{T}$, and $X_{3}={\left[C_{1},C_{2},C_{3},C_{4}\right]}^{T}$. The block matrices are

(A24) $$H_{11}=\left[\begin{array}{cccc} 0 & 1 & 0 & 1\\ \beta_{1} & 0 & \beta_{1} & 0\\ 0 & 0 & 0 & 0\\ 0 & 0 & 0 & 0 \end{array}\right],\;H_{13}=\left[\begin{array}{cccc} 0 & 0 & 0 & 0\\ 0 & 0 & 0 & 0\\ -D_{3}\beta_{3}^{2}s_{3s} & D_{3}\beta_{3}^{2}c_{3s} & D_{3}\beta_{3}^{2}sh_{3s} & D_{3}\beta_{3}^{2}ch_{3s}\\ -D_{3}\beta_{3}^{3}c_{3s} & -D_{3}\beta_{3}^{3}s_{3s} & D_{3}\beta_{3}^{3}ch_{3s} & D_{3}\beta_{3}^{3}sh_{3s} \end{array}\right],$$

(A25) $$\;\;\;\;\;\;\;\;\;\begin{array}{cc} H_{21} & =\left[\begin{array}{cccc} s_{1} & c_{1} & sh_{1} & ch_{1}\\ \beta_{1}c_{1} & -\beta_{1}s_{1} & \beta_{1}ch_{1} & \beta_{1}sh_{1}\\ -D_{1}\beta_{1}^{2}s_{1} & -D_{1}\beta_{1}^{2}c_{1} & D_{1}\beta_{1}^{2}sh_{1} & D_{1}\beta_{1}^{2}ch_{1}\\ -D_{1}\beta_{1}^{3}c_{1} & D_{1}\beta_{1}^{3}s_{1} & D_{1}\beta_{1}^{3}ch_{1} & D_{1}\beta_{1}^{3}sh_{1} \end{array}\right],\\ H_{22} & =\left[\begin{array}{cccc} -s_{2p} & -c_{2p} & -sh_{2p} & -ch_{2p}\\ -\beta_{2}c_{2p} & \beta_{2}s_{2p} & -\beta_{2}ch_{2p} & -\beta_{2}sh_{2p}\\ D_{2}\beta_{2}^{2}s_{2p} & D_{2}\beta_{2}^{2}c_{2p} & -D_{2}\beta_{2}^{2}sh_{2p} & -D_{2}\beta_{2}^{2}ch_{2p}\\ D_{2}\beta_{2}^{3}c_{2p} & -D_{2}\beta_{2}^{3}s_{2p} & -D_{2}\beta_{2}^{3}ch_{2p} & -D_{2}\beta_{2}^{3}sh_{2p} \end{array}\right], \end{array}$$

(A26) $$\begin{array}{cc} H_{32} & =D_{2}\beta_{2}^{3}\left[\begin{array}{cccc} \frac{s_{2m}}{D_{2}\beta_{2}^{3}} & \frac{c_{2m}}{D_{2}\beta_{2}^{3}} & \frac{sh_{2m}}{D_{2}\beta_{2}^{3}} & \frac{ch_{2m}}{D_{2}\beta_{2}^{3}}\\ \frac{c_{2m}}{D_{2}\beta_{2}^{2}} & -\frac{s_{2m}}{D_{2}\beta_{2}^{2}} & \frac{ch_{2m}}{D_{2}\beta_{2}^{2}} & \frac{sh_{2m}}{D_{2}\beta_{2}^{2}}\\ -\frac{s_{2m}}{\beta_{2}} & -\frac{c_{2m}}{\beta_{2}} & \frac{sh_{2m}}{\beta_{2}} & \frac{ch_{2m}}{\beta_{2}}\\ c_{2m} & -s_{2m} & -ch_{2m} & -sh_{2m} \end{array}\right]\\ & \;-D_{2}\beta_{2}^{3}\left[\begin{array}{cccc} 0 & 0 & 0 & 0\\ 0 & 0 & 0 & 0\\ 0 & 0 & 0 & 0\\ \frac{\omega^{2}Ms_{2m}}{D_{2}\beta_{2}^{3}} & \frac{\omega^{2}Mc_{2m}}{D_{2}\beta_{2}^{3}} & \frac{\omega^{2}Msh_{2m}}{D_{2}\beta_{2}^{3}} & \frac{\omega^{2}Mch_{2m}}{D_{2}\beta_{2}^{3}} \end{array}\right],\\ H_{33} & =\left[\begin{array}{cccc} -s_{3m} & -c_{3m} & -sh_{3m} & -ch_{3m}\\ -\beta_{3}c_{3m} & \beta_{3}s_{3m} & -\beta_{3}ch_{3m} & -\beta_{3}sh_{3m}\\ D_{3}\beta_{3}^{2}s_{3m} & D_{3}\beta_{3}^{2}c_{3m} & -D_{3}\beta_{3}^{2}sh_{3m} & -D_{3}\beta_{3}^{2}ch_{3m}\\ -D_{3}\beta_{3}^{3}c_{3m} & D_{3}\beta_{3}^{3}s_{3m} & -D_{3}\beta_{3}^{3}ch_{3m} & -D_{3}\beta_{3}^{3}sh_{3m} \end{array}\right]. \end{array}$$

In the above,

(A27) $$\begin{array}{c} s_{1}=\sin\left(\beta_{1}L_{p}\right),\;c_{1}=\cos\left(\beta_{1}L_{p}\right),\;sh_{1}=\sinh\left(\beta_{1}L_{p}\right),\;ch_{1}=\cosh\left(\beta_{1}L_{p}\right),\\ s_{2p}=\sin\left(\beta_{2}L_{p}\right),\;c_{2p}=\cos\left(\beta_{2}L_{p}\right),\;sh_{2p}=\sinh\left(\beta_{2}L_{p}\right),\;ch_{2p}=\cosh\left(\beta_{2}L_{p}\right),\\ s_{2m}=\sin\left(\beta_{2}L_{m}\right),\;c_{2m}=\cos\left(\beta_{2}L_{m}\right),\;sh_{2m}=\sinh\left(\beta_{2}L_{m}\right),\;ch_{2m}=\cosh\left(\beta_{2}L_{m}\right),\\ s_{3s}=\sin\left(\beta_{3}L_{s}\right),\;c_{3s}=\cos\left(\beta_{3}L_{s}\right),\;sh_{3s}=\sinh\left(\beta_{3}L_{s}\right),\;ch_{3s}=\cosh\left(\beta_{3}L_{s}\right),\\ s_{3m}=\sin\left(\beta_{3}L_{m}\right),\;c_{3m}=\cos\left(\beta_{3}L_{m}\right),\;sh_{3m}=\sinh\left(\beta_{3}L_{m}\right),\;ch_{3m}=\cosh\left(\beta_{3}L_{m}\right). \end{array}$$

A nontrivial solution exists only if the determinant of the coefficient matrix vanishes:

(A28) $$\det\;\left[H(\omega )\right]=0.$$

The smallest positive root $\omega_{1}$ is the first natural circular frequency, and the corresponding natural frequency is $f_{1}=\omega_{1}/\left(2\pi \right)$.

## Appendix B. Generalizability Test of Inverse Design with Random Target Frequencies

To evaluate the generalizability of the proposed TNN-based inverse-design framework within the training domain, 20 sets of target characteristic frequencies were randomly generated within the permissible ranges of the design variables (α∈[0°,49°], $M_{1}$–$M_{5}\in \left[1,15\right]\;g$).

Each set of target frequencies was fed into the trained inverse-design TNN to obtain the corresponding design parameters. Subsequently, the high-accuracy forward-prediction network trained in Section 3.1 (with $R^{2}>0.99$ on the test set) was employed to rapidly predict the corresponding characteristic frequencies, thereby validating the consistency between the inverse-design output and the target frequencies.

As shown in Figure A1a–e, the predicted frequencies agree well with the target frequencies for all five beams across the 20 sets, with $R^{2}$ values consistently above 0.91, indicating that the inverse-design network possesses good generalizability within the training domain.

Furthermore, Figure A1f presents the average prediction error for each design set. The vertical axis represents the mean absolute error, calculated as the average of the absolute differences between the predicted and target values for the five characteristic frequencies in each set:

(A29) $$\mathrm{Mean}\;\mathrm{error}=\frac{1}{5}\sum_{i=1}^{5}\left|F_{\mathrm{pred},i}-F_{\mathrm{target},i}\right|.$$

It can be observed from Figure A1 that the average error is below 1 Hz for most groups, while an increase in error is noted for groups 3, 7, 11, 14, and 19. These groups with higher errors correspond to design parameters located near the boundaries of the training domain (e.g., *α* close to 49°, or multiple $M_{i}$ values simultaneously close to 1 g or 15 g). Such extreme combinations are less represented in the training data, leading to a relative decline in the prediction accuracy of the forward network in these regions, which, in turn, affects the output precision of the inverse-design network.

Nevertheless, the overall $R^{2}$ values remain high, demonstrating that the TNN retains reliable inverse-design capability across most of the design domain and can provide a rapid and effective parameter customization solution for broadband PEH arrays.
